# Sex-Related Disparities in the Prevalence of Depression among Patients Hospitalized with Type 2 Diabetes Mellitus in Spain, 2011–2020

**DOI:** 10.3390/jcm11216260

**Published:** 2022-10-24

**Authors:** Ana Lopez-de-Andres, Rodrigo Jimenez-Garcia, Javier de Miguel-Díez, Valentin Hernández-Barrera, Jose Luis del Barrio, David Carabantes-Alarcon, Jose J. Zamorano-Leon, Concepcion Noriega

**Affiliations:** 1 Department of Public Health and Maternal & Child Health, Faculty of Medicine, Universidad Complutense de Madrid, Instituto de Investigación Sanitaria del Hospital Clínico San Carlos (IdISSC), 28040 Madrid, Spain; 2 Respiratory Department, Hospital General Universitario Gregorio Marañón, Facultad de Medicina, Universidad Complutense de Madrid, Instituto de Investigación Sanitaria Gregorio Marañón (IiSGM), 28007 Madrid, Spain; 3 Preventive Medicine and Public Health Teaching and Research Unit, Health Sciences Faculty, Universidad Rey Juan Carlos, 28922 Alcorcón, Spain; 4 Department of Nursery and Physiotherapy, Faculty of Medicine and Health Sciences, University of Alcalá, Alcalá de Henares, 28801 Madrid, Spain

**Keywords:** type 2 diabetes, depression, sex, in-hospital mortality, hospitalization

## Abstract

(1) Background: Recent reports suggest a decrease in the prevalence of depression among people with diabetes and important sex-differences in the association between these conditions, however data from Spain is sparse. We aim to assess trends in the prevalence of depression and in-hospital outcomes among patients with type 2 diabetes (T2DM) hospitalized (2011–2020) identifying sex-differences. (2) Methods: Using the Spanish national hospital discharge database we analysed the prevalence of depression globally, by sex, and according to the conditions included in the Charlson comorbidity index (CCI). We tested factors associated with the presence of depression and with in-hospital mortality (IHM). Time trends in the prevalence of depression and variables independently associated with IHM were analyzed using multivariable logistic regression. (3) Results: From 2011 to 2020, we identified 5,971,917 hospitalizations of patients with T2DM (5.7% involved depression). The prevalence of depression decreased significantly between 2011 and 2020. The adjusted prevalence of depression was 3.32-fold higher in women than in men (OR 3.32; 95%CI 3.3–3.35). The highest prevalence of depression among men and women with T2DM was found among those who also had a diagnosis of obesity, liver disease, and COPD. Older age, higher CCI, pneumonia, and having been hospitalized in 2020 increased the risk of IHM in patients with T2DM and depression. Obesity was a protective factor for IHM in both sexes, with no differences detected for IHM between men and women. Among patients hospitalized with T2DM, concomitant depression was associated with lower IHM than among patients without depression (depression paradox). (4) Conclusions: The prevalence of depression decreased over time in both sexes. The prevalence of depression was over three-fold higher in women. Female sex and depression were not associated with higher IHM. Based on our results we recommend that clinicians screen regularly for depression in patients with T2DM, particularly women, younger patients, and those with multiple comorbidities.

## 1. Introduction

Diabetes is one of the largest global public health problems, is among the top 10 causes of death globally, and has the second biggest negative total effect on reducing global health adjusted life expectancy worldwide [1]. The Global Burden of Disease (GBD) has demonstrated a large and inexorably increasing burden of diabetes in the world since 1990 [1]. In Spain, according to the GBD, the prevalence and Disability-Adjusted Life Years (DALYs) have increased from 2000 to 2019 from 8.90% to 11.26% and from 3.80% to 4.15% [2].

In year 2019 depressive disorders ranked 13th among the leading causes of burden worldwide with a significant greater burden in females than males [3]. Worldwide, the prevalence of depression among adults has been estimated to be between 15% and 18%, and an increase in the burden of this disease worldwide is projected in the coming decades [4]. In our country the time trend (2000–2019) has shown an increase in the prevalence from 3.57% to 4.24% among men with equivalent figures of 5.72% to 7.71% among women. For both sexes, the DALYs also rose from 2.64% to 3.70% [2].

Like other chronic diseases, type 2 diabetes (T2DM) predisposes to depression [5,6,7]. The presence of complications, including depression, increases the risk of hospitalization among people with T2DM [8]. Depression as a comorbidity of T2DM can alter glycaemic control, reduce adherence to treatment, increase the risk of cardiac complications, health care utilization, costs, and increased mortality risk [9]. In their meta-analysis, Nouwen et al. [10] found that the presence of complications in patients with diabetes increased the likelihood of incident depressive disorder (hazard ratio [HR] 1.14; 95% confidence interval [95% CI]: 1.07–1.21). However, other authors, after adjusting for the presence of comorbidities such as coronary heart disease, found no significant association between T2DM and depression [11,12].

The association between depression and T2DM is thought to be bidirectional [13,14]; however, the role of the factors that modulate this association remains controversial [15]. Depression is associated with inadequate lifestyles (sedentary lifestyle, poor diet, obesity), with increased hypothalamic-pituitary-adrenal axis activity, and with increased levels of stress hormones and proinflammatory cytokines [16]. All these factors may affect insulin resistance and subsequently the presence of T2DM [17].

Recognizing and addressing depression among patients with diabetes is an important step in improving outcomes and reducing the growing burden of diabetes care [18]. Studies conducted in the last decades of the past century and first years of the current century showed that the prevalence of depression among people with diabetes was increasing in our country and elsewhere [17,18,19,20]. However, more recent investigations suggest that a change in the time trend may be taking place [21,22,23]. The lack of recent reports in our country makes necessary to confirm if this new tendency is also happening.

In patients with T2DM and depression, sex differences may play a critical role in the incidence and outcomes of hospitalizations, and meta-analyses have concluded that women with T2DM have a higher prevalence of depression than men with T2DM [24,25]. In 2015, the results of a population study in Spain found that the prevalence of depression was over 2.7 times higher in women with T2DM than in men with T2DM [19]. The probability of being diagnosed with depression in patients with T2DM differs by sex [26,27,28].

There is very little information on the consequences of diabetes-depression comorbidity in the hospital outcomes in Spain [19]. Furthermore, even if no doubt exists regarding the sex-differences in the prevalence of depression among people with diabetes the frequency of other comorbid conditions and how these conditions affect the hospital outcomes among men and women has not been analysed in our country so far.

Therefore, the objective of our study, which was based on national administrative data, was to describe trends in the prevalence of depression in patients with T2DM hospitalized in Spain from 2011 to 2020. Furthermore, we analyzed sex differences in the prevalence of depression among women and men with T2DM according to specific hospital admission diagnoses and the effect of depression on hospital outcomes.

## 2. Materials and Methods

We conducted a population-based cohort study using data from a registry of hospital discharges in Spain (Register of Specialized Care–Basic Minimum Database, RAE-CMBD) collected from 1 January 2011 to 31 December 2020.

The methodological characteristics of the RAE-CMBD have been described elsewhere [29]. Coding in this database was with the International Classification of Diseases (ICD), Ninth Revision, for the period between 2011 and 2015 (ICD-9), and the Tenth Revision (ICD-10) from the year 2016 onwards.

The study population included patients aged ≥35 years with a T2DM code in any diagnostic position (see ICD codes in Table S1). Patients with T1DM were excluded.

To respond to the objective of the study, the study population was stratified according to the presence of ICD codes for depression (Table S1) in any diagnostic position in the RAE-CMBD. All analyses were subsequently stratified according to sex.

To determine the prevalence of depression based on the most frequent hospital admission diagnoses, we used the conditions included in the Charlson Comorbidity Index (CCI) in any diagnostic position, excluding diabetes [30,31]. The presence of codes for obesity and pneumonia was also analysed. Table S1 shows the ICD-9 and ICD-10 codes corresponding to the diagnoses included in our investigation.

Regarding hospital outcomes, we analysed length of hospital stay (LOHS) and in-hospital mortality (IHM).

### 2.1. Statistical Analysis

For all the study years, we calculated the total prevalence of depression in patients with T2DM. The prevalence of depression was stratified by sex, age group, and the abovementioned clinical diagnoses.

Prevalence was calculated by dividing the number of cases of depression in each year by the number of patients with T2DM in each year and subgroup analysed.

The results of the descriptive statistical analysis are expressed as total frequencies with percentages and means with standard deviations for categorical variables and as medians with interquartile range for continuous variables.

The trend was analysed using the Cochran-Mantel-Haenszel statistic or Cochran-Armitage test in the case of categorical variables and a linear regression t test or Jonckheere-Terpstra test in the case of continuous variables.

Categorical variables were compared using the Fisher exact test. Continuous variables were compared using the t test or the Wilcoxon rank sum test, as required.

We used multivariable logistic regression to analyse factors associated with the presence of depression, taking into account the effect of sex. We also identified the variables associated with IHM in men and women with T2DM and depression.

Finally, we used logistic regression to assess the effect of depression on IHM in both men and women with T2DM. Models were constructed for the concomitant clinical conditions included in the CCI, obesity, and pneumonia.

The results were presented using the odds ratio (OR) and 95% CI.

We used version 14 of Stata to perform the statistical analysis (Stata, College Station, TX, USA). Statistical significance was set at *p* < 0.05 (2-tailed).

### 2.2. Ethics

To carry out this study, it was not necessary to request the informed consent of the patients or approval by an ethics committee, since the RAE-CMBD is anonymous and can be requested from the Spanish Ministry of Health [32].

## 3. Results

During the period 2011–2020 in Spain, there were 5,971,917 hospitalizations of patients aged ≥35 years who had an ICD diagnostic code corresponding to T2DM. Of these, 333,226 (5.57%) had an ICD code for depression.

The overall prevalence of depression among patients hospitalized with T2DM in Spain decreased significantly between 2011 and 2020 (5.72% vs. 5.04%; *p* < 0.05); however, it should be noted that the prevalence increased between 2011 and 2015, before decreasing from 2016 to 2020 (Table 1).

Regarding the distribution of the study population according to sex, the proportion of women decreased (71.25% in 2011 vs. 67.89% in 2020; *p* < 0.05), whereas that of men increased (28.75% in 2011 vs. 32.11% in 2020; *p* < 0.05). The mean age of patients with depression, as well as the associated comorbidity based on the CCI, increased significantly throughout the study period (Table 1).

Between 2011 and 2020, the prevalence of depression coded as the primary diagnosis decreased (1.23% in 2011 vs. 0.96% in 2020; *p* < 0.05) (Table 1).

In men with T2DM, the prevalence of depression decreased significantly throughout the study period (2.97% in 2011 vs. 2.75% in 2020). Significant increases were also observed during 2011–2020 for mean age (69.96 years vs. 72.67 years), the presence of associated comorbidity (mean CCI 2.82 vs. 3.3), and a diagnosis of obesity (11.11% vs. 15.25%).

The crude IHM increased significantly between 2011 and 2020 (5.93% vs. 8.89%) (Table 2).

The trend for women with T2DM was similar to that of men regarding prevalence (9.15% in 2011 vs. 8.32% in 2020), mean age (73.42 years vs. 76.51 years), comorbidity (mean CCI 2.35 vs. 2.86), and the presence of obesity (21.74% vs. 24.08%). The crude IHM almost doubled (4.88% vs. 8.58%) over the 10 years analysed (Table 2).

Table 3 shows the prevalence of depression among people with T2DM who also had other specific diagnoses. The highest prevalence of depression among men and women with T2DM was found among those who also had a diagnosis of obesity, liver disease, and COPD. A remarkable finding was the very high prevalence of depression among obese women with diabetes, ranging from 10% to 12% over the study period.

The prevalence of depression decreased significantly between 2011 and 2020, in men and women with T2DM who also had kidney disease, liver disease, cancer, and obesity. However, in patients with peripheral vascular disease and COPD, the prevalence of depression increased significantly.

Throughout the study period and for all specific hospital admission diagnoses, the prevalence of depression was higher in women with T2DM than in men with T2DM (*p* < 0.05).

For all the specific hospital admission diagnoses analysed, IHM was significantly lower in women with T2DM and depression than in women without depression. This association was also found in men with T2DM and depression, except for those with acute myocardial infarction and cancer (Table 4).

Table 5 presents the results of the multivariable analysis to identify the factors associated with the presence of depression and with IHM in men and women with T2DM and depression. Older age and the most recent years of hospital admission (years 2018, 2019, and 2020) were associated with a lower risk of depression. However, the prevalence of depression increased significantly during the years 2013, 2014, and 2015 in both men and women with T2DM. The presence of more comorbid conditions based on the CCI and obesity was associated with a higher probability of a code for depression in both men and women. In the T2DM population, and after adjusting for age and all the comorbid conditions, women were 3.32-fold more likely to have a code for depression in their discharge report than men (OR 3.32; 95%CI 3.3–3.35).

Older age, the presence of comorbidity (CCI), the presence of pneumonia, and having been hospitalized in 2020 increased the risk of IHM in men and women with T2DM and depression (Table 5). However, obesity was a protective factor for IHM in both men and women. Sex was not associated with IHM in patients with depression and T2DM (OR for women 0.97; 95% CI 0.94–1.01).

Finally, after multivariable adjustment by year, age and CCI, among men with T2DM and cerebrovascular disease, kidney disease, and liver disease, the presence of depression was associated with a lower risk of dying in hospital (Table S2). For all the specific hospital admissions analysed (except for cancer) in women with T2DM, the presence of depression had a protective effect on IHM (Table S3).

## 4. Discussion

The results of this nationwide population-based observational study of almost six million patients with T2DM hospitalized in Spain during 2011–2020 reveals several key findings. First, a decrease in the prevalence of depression was observed in men and women with T2DM between 2011 and 2020. Second, the prevalence of depression was 3.32 times higher in women with T2DM than in men. Third, IHM among people with T2DM and depression increased throughout the study period, although IHM was lower in patients with depression than in those without depression. Finally, we found no sex differences in IHM among people hospitalized with T2DM and depression.

Epidemiological studies over the last two decades have reported a global increase in the prevalence of depression in persons with diabetes [20]. As in a previous report, we found an increase in the prevalence of depression in patients hospitalized with T2DM from 2011 to 2015 [19]; however, since 2016, the prevalence of depression has been decreasing. Our results could indicate that care and treatment of people with depression, particularly those with diabetes, is improving. Screening for depression in clinical practice, particularly among people with diabetes, may be a useful first step in identifying patients at high risk of this disease. The change in the time trend of prevalence has been reported in various studies [21,22,23]. In a population-based study in Norway, Bojanic et al. [21] found a general decrease in depressive symptoms in men with diabetes and relatively stable symptoms in women with this condition between 1995-97 and 2017-19, concluding that this change could be explained in part by awareness of these psychological conditions and improvements in treatment. Similarly, a decrease in prevalence was observed in a population-based study of Mexican adults with diabetes (age ≥ 50 years) between 2001 and 2015 [33].

As has been described in the literature, the prevalence of depression is higher in women with diabetes than in men [19,24,25]. In a recent study of 123,232 patients with diabetes mellitus between 1997 and 2014, Deischinger et al. [28] concluded that a diagnosis of depression is more likely in women than in men between age 30 and 69 years (OR 1.37; 95% CI: 1.32–1.43). Various factors seem to contribute to the difference between men and women regarding depression. The prevalence of depression is higher in women owing to biological factors and the fact that the psychological burden of having the disease is greater [27]. Men, on the other hand, are thought to visit their doctor less frequently, potentially leading to underdiagnosis of T2DM and depression. This effect might be even more prominent in a multimorbid condition such as diabetes, which requires more extensive medical care [34].

Furthermore, differences between women and men in psychological reactions following cardiovascular events have been addressed in the literature. In a meta-analysis investigating depression after diagnosis of cardiovascular disease/events, Buckland et al. [35] suggested that women experience a higher level of depression after a coronary event than men.

In our study, obesity was associated with the presence of depression. A recent meta-analysis concluded that persons with T2DM and obesity have a 1.63-fold greater risk of depression than those with T2DM without obesity [36]. A strong relationship between obesity and depression has been described in the literature, especially in women [37]. Vittengl et al. [38] indicate that this relationship can be explained by somatic, behavioural, and psychosocial mechanisms, including physical deterioration, social dysfunction (discrimination based on weight, low participation in social life or little social support), and emotional eating, which are more frequent in women.

Diabetes and depression are chronic diseases in which ageing, and the presence of concomitant medical conditions are crucial factors [13]. However, our results are in line with the literature, showing that depression affects younger people more than older people [6,19,25,39], because younger people find the onset of T2DM more difficult and need a longer period of adaptation to their disease [40].

The presence of depression in persons with diabetes was recently associated with a 2.16-fold increased risk of dying [41]. In our study, IHM increased over time, with particularly high values in 2020 because of COVID-19.

The main risk factors associated with IHM in patients with T2DM diagnosed with depression are older age, comorbidity, the presence of pneumonia, and having been hospitalized in 2020, as reported in the literature [19,41]. However, obesity was a protective factor for IHM, thus confirming the obesity paradox, as described elsewhere [42].

In our study, female sex was not associated with higher IHM in patients with depression, even though depression is more prevalent in women. In a population-based study of 64,177 Norwegian adults, Naicker et al. [26] found that the presence of depression increased the risk of mortality only in men with diabetes (HR 2.47; 95% CI 1.47–4.17) and not in women with diabetes, suggesting that men were more likely to be diagnosed later with depression and treated in more advanced stages of the disease. Consequently, they would present greater functional impairment than women. Sex differences in the prevalence of depressive disorders are well documented, although few studies to date have examined sex differences in outcomes in patients hospitalized with depression.

Finally, we found that among men and women with T2DM who also experienced depression, the risk of dying in hospital was similar to or even lower than among men and women without depression. This unexpected result has also been reported elsewhere [43,44,45]. In their population-based study comparing 38,537 diabetic patients with depression and 154,148 diabetic patients without depression, Wu et al. [43] concluded that there were no statistically significant differences in mortality from cardiovascular diseases. Patients with depression adhered slightly better to antidiabetic medication and slightly more underwent screening tests, thus potentially explaining the authors’ results. Pino et al. [44] studied patients in the general population hospitalized for ST-elevation myocardial infarction and found that, paradoxically, the probability of dying in hospital was lower among patients with a clinically co-occurring depression and/or anxiety than those without. As an explanation for this “depression paradox”, the authors suggested potentially underdiagnosed mental health issues surrounding major cardiovascular events, and indeed, chronic disease as a whole. Depression has also been associated with lower in-hospital mortality in patients undergoing colorectal surgery [45]. Another possible reason is that T2DM patients with depression are hospitalized with less severe acute or chronic conditions, thus increasing their probability of surviving. More studies are needed to understand the factors underlying this paradox and whether information or selection bias is responsible for the association.

The strengths of our study are the use of a national population database (RAE-CMBD), the 10-year study period, and the fact that our methodology has been used elsewhere [19]. However, our study is also subject to a series of limitations. The RAE-CMBD collects practically all hospitalizations in Spain; however, it is an administrative database and does not collect all the variables included in the clinical history. Therefore, we have no data on disease severity, glycaemic control, disease duration, or medication for diabetes or depression.

The decreased frequency of depression as of 2016 could be explained by the change in coding in the RAE-CMBD. Consequently, the results should be interpreted with caution. In addition, given that the RAE-CMBD provides anonymous patient data, we cannot know whether a patient has been admitted more than once during the same year or whether he/she has been transferred to another hospital, in which case he/she could appear twice. However, the use of hospital discharge records and administrative databases for the diagnosis of psychiatric illnesses, including depression, has been shown to be sufficiently sensitive and specific for epidemiological investigations [46,47]. Finally, in year 2020 the COVID19 pandemic had a very important impact in the Spanish health services and hospitals were collapsed by patients with this infection. This may have resulted in underdiagnose of depression that year. However, the decreasing trend in the prevalence of depression among patients with T2DM was also observed in the years immediately before 2020.

## 5. Conclusions

The prevalence of depression in men and women with T2DM decreased between 2011 and 2020. Our data highlight major sex differences, indicating that the prevalence of depression is more than three times higher in women than in men. However, female sex and depression are not associated with higher IHM than male sex. Older age, associated comorbidity, the presence of pneumonia, and having been hospitalized in 2020 are predictors of IHM in men and women with T2DM and depression, with obesity being a protective factor. IHM is lower in patients with T2DM and depression than in patients without depression. Future studies should analyse the possible existence of a depression paradox for in-hospital mortality among people with diabetes.

Based on our results we recommend that clinicians screen regularly for depression in patients with T2DM, particularly women, younger patients, and those with multiple comorbidities.

## Figures and Tables

**Table 1:** Characteristics of the patients hospitalized with type 2 diabetes suffering concomitant depression according to year (2011–2020).

	2011	2012	2013	2014	2015	2016	2017	2018	2019	2020
Depression, n	31,101	32,236	34,471	36,401	37,684	30,669	34,680	32,218	33,475	30,291
Depression, prevalence (%) *	5.72	5.79	6.05	6.19	6.21	5.41	5.57	4.94	5.03	5.04
Men, n (%) *	8941 (28.75)	9260 (28.73)	10,132 (29.39)	10,323 (28.36)	11,042 (29.3)	9500 (30.98)	10,477 (30.21)	10,026 (31.12)	10,572 (31.58)	9727 (32.11)
Women, n (%) *	22,160 (71.25)	22,976 (71.27)	24,339 (70.61)	26,078 (71.64)	26,642 (70.7)	21,169 (69.02)	24,203 (69.79)	22,192 (68.88)	22,903 (68.42)	20,564 (67.89)
Age, mean (SD) *	72.42 (11.41)	72.81 (11.36)	72.9 (11.43)	73.08 (11.44)	73.55 (11.45)	74.14 (11.39)	74.51 (11.34)	74.89 (11.26)	75.12 (11.22)	75.28 (11.28)
CCI, mean (SD) *	2.49 (1.65)	2.51 (1.65)	2.55 (1.69)	2.55 (1.69)	2.55 (1.65)	2.8 (1.8)	2.84 (1.81)	2.9 (1.87)	2.98 (1.91)	3 (1.94)
Depression as first diagnosis, n (%) *	382 (1.23)	398 (1.23)	382 (1.11)	372 (1.02)	404 (1.07)	344 (1.12)	344 (0.99)	382 (1.19)	323 (0.96)	291 (0.96)

CCI: Charlson Comorbidity Index. * *p* < 0.05 (time trend analysis).

**Table 2:** Prevalence of depression, distribution by age and clinical characteristics and in-hospital outcomes among men and women hospitalized with type 2 diabetes in Spain 2011–2020.

		2011	2012	2013	2014	2015	2016	2017	2018	2019	2020
Men	Depression, n (Prevalence) *	8941 (2.97)	9260 (2.99)	10,132 (3.16)	10,323 (3.12)	11,042 (3.21)	9500 (2.94)	10,477 (2.93)	10,026 (2.65)	10,572 (2.73)	9727 (2.75)
	Age, mean (SD) *	69.96 (11.73)	70.16 (11.70)	70.17 (11.74)	70.51 (11.87)	70.92 (11.78)	71.28 (11.83)	71.77 (11.60)	72.17 (11.54)	72.63 (11.34)	72.67 (11.48)
	35–59 year, n (%) *	1824 (3.72)	1810 (3.72)	2068 (4.14)	2061 (4.09)	1974 (3.90)	1708 (3.68)	1685 (3.44)	1513 (3.01)	1491 (2.97)	1413 (3.02)
	60–69 year, n (%) *	2177 (2.94)	2322 (3.03)	2552 (3.19)	2522 (3.08)	2766 (3.35)	2218 (2.88)	2612 (3.07)	2413 (2.70)	2449 (2.70)	2182 (2.64)
	70–79 year, n (%) *	2833 (2.74)	2922 (2.81)	3013 (2.86)	3025 (2.80)	3299 (2.96)	2851 (2.74)	3088 (2.71)	3040 (2.46)	3352 (2.57)	3110 (2.63)
	≥80 year, n (%) *	2107 (2.82)	2206 (2.73)	2499 (2.94)	2715 (2.98)	3003 (3.03)	2723 (2.84)	3092 (2.83)	3060 (2.65)	3280 (2.82)	3022 (2.85)
	CCI, mean (SD) *	2.82 (1.82)	2.83 (1.80)	2.86 (1.83)	2.86 (1.82)	2.83 (1.78)	3.14 (1.96)	3.18 (1.97)	3.23 (2)	3.29 (2.06)	3.3 (2.09)
	Obesity, n (%) *	993 (11.11)	1001 (10.81)	1167 (11.52)	1275 (12.35)	1440 (13.04)	1316 (13.85)	1507 (14.38)	1469 (14.65)	1550 (14.66)	1483 (15.25)
	LHS, median (IQR)	6 (8)	6 (8)	6 (8)	6 (7)	6 (7)	6 (8)	6 (8)	6 (8)	6 (8)	6 (8)
	IHM, n (%) *	530 (5.93)	526 (5.68)	583 (5.75)	542 (5.25)	626 (5.67)	561 (5.91)	691 (6.60)	687 (6.85)	736 (6.96)	865 (8.89)
Women	Depression, n (Prevalence) *	22,160 (9.15)	22,976 (9.33)	24,339 (9.75)	26,078 (10.16)	26,642 (10.14)	21,169 (8.71)	24,203 (9.13)	22,192 (8.11)	22,903 (8.23)	20,564 (8.32)
	Age, mean (SD) *	73.42 (11.12)	73.88 (11.04)	74.03 (11.11)	74.1 (11.10)	74.64 (11.14)	75.42 (10.95)	75.7 (11.02)	76.13 (10.91)	76.27 (10.97)	76.51 (10.97)
	35–59 year, n (%) *	2802 (12.14)	2732 (12.03)	2831 (12.14)	2981 (12.33)	2858 (12.14)	2084 (9.62)	2271 (9.87)	1927 (8.26)	1936 (7.93)	1695 (7.81)
	60–69 year, n (%) *	4315 (11.21)	4530 (11.60)	4727 (11.81)	5129 (12.66)	4990 (12.50)	3638 (10.19)	4237 (10.78)	3709 (9.26)	3828 (9.51)	330 0 (9.24)
	70–79 year, n (%) *	7718 (9.65)	7675 (9.78)	8032 (10.36)	8351 (10.80)	8458 (11.05)	6616 (9.46)	7278 (9.97)	6683 (8.79)	6921 (8.81)	6286 (9.14)
	≥80 year, n (%) *	7325 (7.28)	8039 (7.58)	8749 (8.04)	9617 (8.39)	10,336 (8.42)	8831 (7.64)	10,417 (8.02)	9873 (7.36)	10,218 (7.57)	9283 (7.67)
	CCI, mean (SD) *	2.35 (1.56)	2.38 (1.57)	2.43 (1.61)	2.42 (1.62)	2.43 (1.59)	2.65 (1.70)	2.69 (1.72)	2.75 (1.79)	2.83 (1.81)	2.86 (1.86)
	Obesity, n (%) *	4817 (21.74)	4858 (21.14)	5427 (22.30)	5772 (22.13)	5957 (22.36)	5093 (24.06)	5730 (23.67)	5010 (22.58)	5567 (24.31)	4951 (24.08)
	LHS, median (IQR)	6 (8)	6 (7)	6 (7)	6 (7)	6 (7)	6 (7)	6 (7)	6 (7)	6 (7)	6 (7)
	IHM, n (%) *	1081 (4.88)	1185 (5.16)	1209 (4.97)	1266 (4.85)	1423 (5.34)	1221 (5.77)	1473 (6.09)	1398 (6.3)	1467 (6.41)	1764 (8.58)

Prevalence: Prevalence of depression among patients hospitalized with type 2 diabetes. CCI: Charlson Comorbidity Index. LHS: length of hospital stay. IQR inter quartile range. IHM: in-hospital mortality. * *p* < 0.05 (time trend analysis).

**Table 3:** Prevalence of depression among men and women hospitalized with type 2 diabetes, according to the presence of selected concomitant conditions in Spain from year 2011 to year 2020.

		2011	2012	2013	2014	2015	2016	2017	2018	2019	2020
Acute myocardial infarction, n (%)	Men *	697 (2.5)	620 (2.25)	724 (2.65)	664 (2.48)	703 (2.6)	870 (2.48)	969 (2.36)	1032 (2.25)	1117 (2.28)	998 (2.22)
	Women *	862 (6.8)	834 (6.69)	846 (6.79)	948 (8.02)	866 (7.61)	957 (6.74)	1219 (7.58)	1175 (6.76)	1237 (6.71)	1176 (7)
Congestive heart failure, n (%)	Men *	1212 (2.41)	1296 (2.46)	1427 (2.57)	1466 (2.57)	1568 (2.57)	1656 (2.73)	1829 (2.69)	1853 (2.48)	1971 (2.51)	1770 (2.52)
	Women *	4154 (7.84)	4278 (7.74)	4545 (8.16)	4734 (8.24)	4918 (8.21)	4725 (8.17)	5432 (8.51)	5051 (7.53)	5433 (7.76)	4612 (7.65)
Peripheral vascular disease, n (%)	Men *	789 (2.25)	854 (2.37)	947 (2.43)	961 (2.44)	977 (2.41)	1022 (2.63)	1145 (2.64)	1153 (2.51)	1219 (2.5)	1093 (2.39)
	Women *	808 (6.69)	821 (6.77)	916 (7.21)	929 (7.48)	924 (7.25)	835 (7.46)	951 (7.73)	822 (6.76)	951 (7.05)	861 (6.9)
Cerebrovascular disease, n (%)	Men	1142 (3.48)	1251 (3.65)	1296 (3.69)	1287 (3.54)	1383 (3.73)	1282 (3.73)	1453 (3.77)	1382 (3.43)	1512 (3.59)	1345 (3.49)
	Women *	2415 (9.05)	2571 (9.6)	2716 (9.86)	2828 (10.26)	2816 (10.18)	2325 (9.18)	2751 (9.97)	2280 (8.19)	2622 (8.9)	2298 (8.69)
COPD, n (%)	Men*	2286 (3.04)	2443 (3.17)	2581 (3.29)	2734 (3.37)	2887 (3.4)	2535 (3.34)	2868 (3.42)	2607 (3)	2808 (3.19)	2389 (3.2)
	Women *	3528 (9.95)	3821 (10.26)	4253 (10.97)	4489 (10.89)	4755 (11.08)	3829 (11.75)	4339 (11.88)	3600 (10.09)	3834 (10.29)	3258 (10.37)
Renal disease, n (%)	Men*	1162 (2.33)	1257 (2.3)	1528 (2.56)	1574 (2.46)	1716 (2.45)	1951 (2.77)	2279 (2.81)	2222 (2.5)	2358 (2.5)	2254 (2.6)
	Women *	2435 (6.57)	2725 (6.77)	3089 (7.1)	3404 (7.23)	3785 (7.42)	3979 (7.77)	4794 (8.05)	4612 (7.27)	4934 (7.37)	4541 (7.5)
Liver disease, n (%)	Men*	805 (3.02)	902 (3.29)	1016 (3.47)	1043 (3.4)	1114 (3.52)	966 (3.33)	1131 (3.4)	1143 (3.12)	1173 (2.97)	1072 (2.91)
	Women *	1376 (10.28)	1497 (10.54)	1592 (10.8)	1857 (11.79)	1854 (11.61)	1480 (10.58)	1895 (11.63)	1758 (10.03)	1897 (9.95)	1817 (10.23)
Cancer, n (%)	Men *	1440 (2.72)	1421 (2.58)	1577 (2.74)	1574 (2.64)	1630 (2.68)	1536 (2.62)	1691 (2.56)	1563 (2.23)	1685 (2.3)	1528 (2.31)
	Women *	2090 (8.45)	2125 (8.25)	2368 (8.95)	2489 (9.07)	2577 (9.45)	2027 (7.77)	2287 (7.88)	2227 (7.34)	2325 (7.33)	2100 (7.26)
Obesity, n (%)	Men*	993 (3.41)	1001 (3.21)	1167 (3.42)	1275 (3.51)	1440 (3.74)	1316 (3.56)	1507 (3.51)	1469 (3.12)	1550 (3.1)	1483 (3.03)
	Women *	4817 (12.15)	4858 (11.73)	5427 (12.55)	5772 (12.72)	5957 (12.79)	5093 (11.54)	5730 (11.5)	5010 (9.94)	5567 (10.4)	4951 (10.26)
Pneumonia, n (%)	Men	735 (3.13)	808 (3.28)	818 (3.43)	841 (3.21)	974 (3.37)	719 (3.43)	779 (3.24)	763 (2.96)	789 (3.13)	644 (3.08)
	Women *	1318 (8.34)	1460 (8.77)	1409 (8.96)	1604 (9.19)	1921 (9.7)	1201 (9.34)	1381 (9.48)	1359 (8.7)	1295 (8.61)	1014 (8.67)

COPD: chronic obstructive pulmonary disease. Liver disease: moderate or severe. * *p* < 0.05 (time trend analysis).

**Table 4:** In-hospital mortality among men and women hospitalized with type 2 diabetes, according to selected concomitant condition and to the presence of depression in Spain for the period 2011–2020.

	IHM Men			IHM Women		
	Without Depression	With Depression	*p*-Value	Without Depression	With Depression	*p*-Value
Acute myocardial infarction, n (%)	25,562 (7.43)	587 (6.99)	0.132	14,672 (10.99)	904 (8.93)	<0.001
Congestive heart failure, n (%)	62,701 (10.23)	1525 (9.5)	0.003	64,516 (11.68)	4220 (8.81)	<0.001
Peripheral vascular disease, n (%)	28,165 (7)	656 (6.46)	0.034	11,607 (10.1)	700 (7.94)	<0.001
Cerebrovascular disease, n (%)	37,223 (10.45)	1104 (8.28)	<0.001	32,625 (13.19)	2352 (9.18)	<0.001
COPD, n (%)	58,614 (7.52)	1737 (6.65)	<0.001	23,433 (7.11)	1877 (4.73)	<0.001
Renal disease, n (%)	66,215 (9.44)	1535 (8.39)	<0.001	53,524 (11.1)	3254 (8.5)	<0.001
Liver disease, n (%)	25,559 (8.23)	636 (6.14)	<0.001	12,184 (8.6)	990 (5.82)	<0.001
Cancer, n (%)	76,108 (12.59)	1973 (12.61)	0.946	34,828 (13.65)	2866 (12.67)	<0.001
Obesity, n (%)	16,404 (4.29)	474 (3.59)	<0.001	23,860 (5.83)	2218 (4.17)	<0.001
Pneumonia, n (%)	36,745 (15.56)	1134 (14.41)	0.006	24,602 (17.41)	1760 (12.61)	<0.001

IHM: in-hospital mortality. COPD: chronic obstructive pulmonary disease.

**Table 5:** Multivariate analysis of the factors associated with the presence of depression among men and women hospitalized with type 2 diabetes and factors associated with in-hospital mortality among patients with type 2 diabetes and concomitant depression., Spain 2011–2020.

	Presence of Depression			IHM of Patients with T2DM and Depression		
	Men	Women	Both Sexes	Men	Women	Both Sexes
	OR (95%CI)	OR (95%CI)	OR (95%CI)	OR (95%CI)	OR (95%CI)	OR (95%CI)
Year 2011	1	1	1	1	1	1
Year 2012	1.01 (0.98–1.04)	1.03 (1.01–1.05)	1.02 (1–1.04)	0.95 (0.83–1.07)	1.03 (0.94–1.12)	1 (0.93–1.07)
Year 2013	1.07 (1.04–1.1)	1.08 (1.06–1.1)	1.07 (1.06–1.09)	0.95 (0.84–1.08)	0.97 (0.89–1.05)	0.96 (0.9–1.03)
Year 2014	1.05 (1.02–1.08)	1.13 (1.11–1.15)	1.11 (1.09–1.12)	0.86 (0.75–0.97)	0.93 (0.86–1.01)	0.91 (0.85–0.97)
Year 2015	1.09 (1.06–1.12)	1.13 (1.11–1.16)	1.12 (1.1–1.14)	0.92 (0.81–1.03)	1 (0.92–1.09)	0.97 (0.91–1.04)
Year 2016	1 (0.97–1.03)	0.96 (0.94–0.98)	0.97 (0.96–0.99)	0.87 (0.77–0.98)	1 (0.91–1.09)	0.95 (0.89–1.02)
Year 2017	1 (0.97–1.02)	1.02 (1–1.04)	1.01 (0.99–1.03)	0.97 (0.86–1.1)	1.03 (0.95–1.12)	1.01 (0.95–1.08)
Year 2018	0.9 (0.87–0.92)	0.9 (0.88–0.91)	0.9 (0.88–0.91)	0.98 (0.87–1.1)	1.01 (0.93–1.1)	1 (0.94–1.07)
Year 2019	0.93 (0.9–0.96)	0.91 (0.89–0.93)	0.92 (0.9–0.93)	0.96 (0.86–1.09)	1.01 (0.93–1.1)	1 (0.93–1.07)
Year 2020	0.94 (0.91–0.97)	0.92 (0.91–0.94)	0.93 (0.91–0.94)	1.3 (1.15–1.45)	1.4 (1.3–1.52)	1.37 (1.28–1.46)
Age, 35–59 years	1	1	1	1	1	1
Age, 60–69 years	0.84 (0.82–0.85)	1.08 (1.06–1.1)	0.98 (0.96–0.99)	1.67 (1.48–1.88)	1.41 (1.26–1.58)	1.52 (1.4–1.65)
Age, 70–79 years	0.78 (0.76–0.79)	0.98 (0.96–0.99)	0.9 (0.89–0.91)	2.31 (2.07–2.58)	2.19 (1.98–2.42)	2.23 (2.07–2.41)
Age, ≥80 year	0.82 (0.8–0.83)	0.79 (0.78–0.8)	0.78 (0.77–0.79)	4.02 (3.61–4.48)	4.39 (3.98–4.83)	4.29 (3.99–4.61)
CCI	1.13 (1.11–1.16)	1.03 (1.01–1.05)	1.07 (1.06–1.09)	1.3 (1.28–1.31)	1.34 (1.33–1.35)	1.33 (1.32–1.34)
Obesity	1.14 (1.12–1.16)	1.33 (1.32–1.35)	1.29 (1.27–1.3)	0.66 (0.6–0.73)	0.78 (0.74–0.82)	0.75 (0.72–0.79)
Pneumonia	0.97 (0.94–1.02)	0.95 (0.91–1.01)	0.96 (0.93–1.01)	2.48 (2.31–2.66)	2.37 (2.24–2.51)	2.41 (2.31–2.52)
Women			3.32 (3.3–3.35)			0.97 (0.94–1.01)

IHM: In-Hospital Mortality. T2DM: Type 2 diabetes. CCI: Charlson Comorbidity Index. OR: Odds Ratio.CI: Confidence interval.

## Data Availability

According to the contract signed with the Spanish Ministry of Health and Social Services, which provided access to the databases from the Spanish National Hospital Database (RAE-CMBD, Registro de Actividad de Atención Especializada. Conjunto Mínimo Básico de Datos, Registry of Specialized Health Care Activities. Minimum Basic Data Set), we cannot share the databases with any other investigator, and we have to destroy the databases once the investigation has concluded. Consequently, we cannot upload the databases to any public repository. However, any investigator can apply for access to the databases by filling out the questionnaire available at https://www.sanidad.gob.es/estadEstudios/estadisticas/estadisticas/estMinisterio/SolicitudCMBD.htm. (accessed on 20 October 2022). All other relevant data are included in the paper.

## Supplementary Materials

The following supporting information can be downloaded at: https://www.mdpi.com/article/10.3390/jcm11216260/s1, Table S1: Diagnosis analyzed with their corresponding ICD-9-CM and ICD10 codes; Table S2: Multivariate analysis of the factors associated with in-hospital mortality in men with type 2 diabetes and selected concomitant conditions in Spain, 2011–2020; Table S3: Multivariate analysis of the factors associated with in-hospital mortality in women with type 2 diabetes and selected concomitant conditions in Spain, 2011–2020.
