# Supplementary material for: Sex-Related Disparities in the Prevalence of Depression among Patients Hospitalized with Type 2 Diabetes Mellitus in Spain, 2011–2020
Source: J Clin Med. 2022 Oct 24;11(21):6260. doi: 10.3390/jcm11216260 (PMC9654856; doi:10.3390/jcm11216260)
Supplement: Supplementary file 1 [file jcm-11-06260-s001.zip › jcm-1970434-supplementary.pdf]

Table S1. Diagnosis analyzed with their corresponding ICD-9-CM and ICD10 codes.

DIAGNOSIS	ICD-9-CM codes	ICD-10 codes
Type 2 diabetes	250.x0; 250.x2	E11.x
Depression	300.4; 301.12; 309.1; 311	F34.1, F43.21; F32.9
Acute Myocardial Infarction	410, 412	I21, I22, I252
Congestive Heart Failure	398.91,402.01,402.11,402.91,404.01, 404.03,404.11,404.13,404.91,404.93,425.4–425.9, 428.x	I50
Peripheral Vascular Disease	0.93.0,473.3,440.x,441.x,443.1-443.9,447.1,557.1,557.9,V43.4	I71, I790, I739, R02, Z958, Z959
Cerebrovascular Disease	362.34, 430.x–438.x	I60, I61, I62, I63, I65, I66,G450, G451, G452, G458, G459, G46, I64, G454, I670, I671, I672, I674, I675, I676, I677 I678, I679, I681, I682, I688, I69
COPD	416.8, 416.9, 490.x–505.x, 506.4, 508.1, 508.8	I27.8, I27.9, J40.x–J47.x, J60.x–J67.x, J68.4, J70.1, J70.3
Renal Disease	403.01, 403.11, 403.91, 404.02, 404.03, 404.12, 404.13, 404.92, 404.93, 582.x, 583.0 - 583.7, 585.x, 586.x, 588.0, V42.0, V45.1, V56.x	I12.0, I13.1, N03.2–N03.7, N05.2–N05.7, N18.x, N19.x, N25.0, Z49.0–Z49.2, Z94.0, Z99.2
Liver Disease	070.22, 070.23, 070.32, 070.33, 070.44, 070.54, 070.6, 070.9, 570.x, 571.x, 573.3, 573.4, 573.8, 573.9, V42.7; 456.0 - 456.2, 572.2-572.8	B18.x, K70.0 - K70.3, K70.9, K71.3 - K71.5, K71.7, K73.x, K74.x, K76.0, K76.2 - K76.4, K76.8, K76.9, Z94.4, I85.0, I85.9, I86.4, I98.2, K70.4, K71.1, K72.1, K72.9, K76.5, K76.6, K76.7
Cancer	140.x–172.x, 174.x–195.8, 200.x–208.x, 238.6, 196.x–199.x	C00.x - C26.x, C30.x - C34.x, C37.x - C41.x, C43.x, C45.x - C58.x, C60.x - C76.x, C81.x - C85.x, C88.x, C90.x - C97.x, C77.x - C80.x
Obesity	278.0x, 649.1x ,V85.3, V85.4	E66.09, E66.1 E66.3, E66.8 E66.9 E66.2, E66.01
Pneumonia	997.31, 507.xx, 480.xx-488.xx	J12 to J18¸ J12-J18, J95.851

Table S2. Multivariate analysis of the factors associated with in-hospital mortality in men with type 2 diabetes and selected concomitant conditions in Spain, 2011-2020.

	**Acute myocardial infarction**	**Congestive heart failure**	**Peripheral vascular disease**	**Cerebrovascular disease**	**COPD**	**Renal disease**	**Liver disease**	**Cancer**	**Obesity**	**Pneumonia**
	**OR(95%CI)**	**OR(95%CI)**	**OR(95%CI)**	**OR(95%CI)**	**OR(95%CI)**	**OR(95%CI)**	**OR(95%CI)**	**OR(95%CI)**	**OR(95%CI)**	**OR(95%CI)**
Year 2011	1	1	1	1	1	1	1	1	1	1
Year 2012	0.99(0.92-1.05)	0.96(0.92-1)	1(0.94-1.06)	0.95(0.91-1)	0.96(0.93-1)	0.95(0.91-0.99)	0.95(0.89-1.01)	0.96(0.93-1)	0.97(0.89-1.06)	0.97(0.93-1.02)
Year 2013	0.9(0.84-0.96)	0.89(0.86-0.93)	0.91(0.86-0.97)	0.87(0.83-0.92)	0.9(0.87-0.94)	0.86(0.83-0.9)	0.86(0.81-0.91)	0.92(0.89-0.95)	0.8(0.74-0.88)	0.92(0.88-0.97)
Year 2014	0.89(0.84-0.95)	0.89(0.85-0.92)	0.89(0.84-0.95)	0.88(0.84-0.93)	0.87(0.84-0.9)	0.89(0.85-0.92)	0.84(0.79-0.89)	0.88(0.85-0.91)	0.82(0.75-0.89)	0.9(0.86-0.95)
Year 2015	0.93(0.87-0.99)	0.89(0.86-0.93)	0.9(0.85-0.95)	0.89(0.84-0.93)	0.91(0.87-0.94)	0.9(0.86-0.93)	0.85(0.8-0.9)	0.89(0.86-0.92)	0.89(0.82-0.96)	0.9(0.86-0.94)
Year 2016	0.84(0.79-0.89)	0.88(0.85-0.92)	0.88(0.83-0.93)	0.88(0.83-0.92)	0.89(0.86-0.93)	0.89(0.85-0.92)	0.78(0.74-0.83)	0.9(0.87-0.94)	0.91(0.84-0.98)	0.69(0.65-0.73)
Year 2017	0.81(0.76-0.86)	0.87(0.84-0.91)	0.86(0.81-0.91)	0.87(0.83-0.92)	0.87(0.84-0.91)	0.89(0.85-0.92)	0.77(0.73-0.82)	0.89(0.86-0.92)	0.94(0.88-1.02)	0.67(0.64-0.71)
Year 2018	0.78(0.74-0.83)	0.88(0.84-0.91)	0.88(0.83-0.93)	0.9(0.86-0.94)	0.86(0.83-0.89)	0.86(0.83-0.9)	0.73(0.69-0.78)	0.85(0.82-0.88)	0.86(0.8-0.93)	0.66(0.62-0.69)
Year 2019	0.74(0.7-0.79)	0.84(0.81-0.87)	0.82(0.77-0.87)	0.83(0.79-0.87)	0.82(0.79-0.85)	0.83(0.8-0.86)	0.67(0.63-0.71)	0.83(0.81-0.86)	0.83(0.77-0.9)	0.61(0.58-0.64)
Year 2020	0.94(0.89-1)	1.08(1.04-1.12)	1.05(0.99-1.11)	1.01(0.97-1.06)	1.09(1.05-1.13)	1.09(1.05-1.13)	0.82(0.77-0.86)	0.87(0.84-0.91)	1.25(1.17-1.35)	0.83(0.78-0.87)
Age, 35-59 years	1	1	1	1	1	1	1	1	1	1
Age, 60-69 years	1.5(1.41-1.6)	1.24(1.18-1.3)	1.41(1.33-1.51)	1.25(1.18-1.32)	1.4(1.33-1.47)	1.4(1.33-1.48)	1.28(1.23-1.33)	1.02(0.99-1.06)	1.36(1.28-1.44)	1.17(1.1-1.24)
Age, 70-79 years	2.23(2.1-2.37)	1.74(1.67-1.82)	2.08(1.95-2.21)	1.74(1.65-1.83)	1.91(1.82-2.01)	2.05(1.96-2.16)	1.65(1.59-1.71)	1.18(1.15-1.22)	1.93(1.83-2.04)	1.43(1.35-1.51)
Age, ≥80 year	4.13(3.89-4.38)	3.04(2.91-3.18)	3.89(3.66-4.14)	2.99(2.84-3.15)	3.34(3.19-3.5)	3.84(3.67-4.03)	2.55(2.44-2.66)	1.74(1.68-1.79)	3.71(3.52-3.92)	2.3(2.18-2.42)
CCI	1.25(1.25-1.26)	1.2(1.19-1.2)	1.27(1.26-1.28)	1.18(1.18-1.19)	1.28(1.28-1.29)	1.25(1.25-1.26)	1.32(1.31-1.32)	1.3(1.29-1.3)	1.32(1.32-1.33)	1.18(1.17-1.18)
Depression	0.99(0.9-1.08)	0.94(0.89-1)	0.94(0.87-1.02)	0.81(0.76-0.87)	0.98(0.93-1.03)	0.9(0.85-0.95)	0.83(0.77-0.91)	1.03(0.98-1.08)	0.91(0.83-1)	0.95(0.89-1.01)

COPD: chronic obstructive pulmonary disease. CCI: Charlson Comorbidity Index. Excluding diabetes and the condition analysed. OR: Odds Ratio.CI: Confidence interval.

Table S3. Multivariate analysis of the factors associated with in-hospital mortality in women with type 2 diabetes and selected concomitant conditions in Spain, 2011-2020.

	**Acute myocardial infarction**	**Congestive heart failure**	**Peripheral vascular disease**	**Cerebrovascular disease**	**COPD**	**Renal disease**	**Liver disease**	**Cancer**	**Obesity**	**Pneumonia**
	**OR(95%CI)**	**OR(95%CI)**	**OR(95%CI)**	**OR(95%CI)**	**OR(95%CI)**	**OR(95%CI)**	**OR(95%CI)**	**OR(95%CI)**	**OR(95%CI)**	**OR(95%CI)**
Year 2011	1	1	1	1	1	1	1	1	1	1
Year 2012	1(0.92-1.08)	1.01(0.97-1.05)	1.02(0.93-1.11)	1.02(0.93-1.11)	0.99(0.93-1.05)	0.99(0.94-1.03)	0.99(0.91-1.08)	0.93(0.88-0.98)	0.98(0.92-1.04)	0.98(0.93-1.04)
Year 2013	0.96(0.89-1.04)	0.96(0.93-1)	0.94(0.87-1.03)	0.94(0.87-1.03)	0.92(0.87-0.98)	0.93(0.88-0.97)	0.93(0.86-1.02)	0.89(0.85-0.94)	0.91(0.85-0.97)	0.93(0.88-0.98)
Year 2014	0.9(0.83-0.98)	0.94(0.9-0.97)	0.97(0.89-1.06)	0.97(0.89-1.06)	0.95(0.89-1)	0.89(0.85-0.93)	0.93(0.86-1.01)	0.89(0.85-0.94)	0.88(0.82-0.93)	0.9(0.86-0.96)
Year 2015	0.95(0.88-1.04)	0.97(0.93-1.01)	0.96(0.88-1.04)	0.96(0.88-1.04)	0.93(0.88-0.99)	0.92(0.88-0.96)	0.91(0.84-0.99)	0.88(0.83-0.92)	0.89(0.84-0.95)	0.87(0.82-0.92)
Year 2016	0.84(0.78-0.91)	0.95(0.91-0.99)	0.9(0.82-0.98)	0.9(0.82-0.98)	0.89(0.84-0.95)	0.92(0.88-0.96)	0.79(0.73-0.87)	0.89(0.85-0.94)	0.96(0.91-1.02)	0.67(0.62-0.71)
Year 2017	0.87(0.81-0.94)	0.98(0.94-1.02)	0.95(0.87-1.03)	0.95(0.87-1.03)	0.88(0.83-0.93)	0.95(0.91-0.99)	0.75(0.69-0.82)	0.91(0.87-0.96)	0.99(0.93-1.05)	0.65(0.61-0.69)
Year 2018	0.83(0.77-0.89)	0.96(0.92-0.99)	0.95(0.87-1.04)	0.95(0.87-1.04)	0.86(0.81-0.91)	0.92(0.88-0.96)	0.74(0.68-0.81)	0.87(0.83-0.92)	0.95(0.9-1.01)	0.59(0.56-0.63)
Year 2019	0.77(0.72-0.83)	0.94(0.9-0.97)	0.85(0.79-0.93)	0.85(0.79-0.93)	0.86(0.81-0.91)	0.88(0.85-0.92)	0.68(0.63-0.74)	0.86(0.81-0.9)	0.9(0.84-0.95)	0.57(0.53-0.6)
Year 2020	0.91(0.85-0.98)	1.17(1.12-1.21)	1.01(0.93-1.1)	1.01(0.93-1.1)	1.13(1.06-1.2)	1.12(1.08-1.17)	0.83(0.77-0.9)	0.9(0.86-0.95)	1.26(1.19-1.33)	0.76(0.72-0.81)
Age, 35-59 years	1	1	1	1	1	1	1	1	1	1
Age, 60-69 years	1.76(1.53-2.03)	1.13(1.04-1.23)	1.78(1.53-2.06)	1.78(1.53-2.06)	1.48(1.36-1.62)	1.4(1.28-1.52)	1.47(1.36-1.6)	1.13(1.07-1.19)	1.67(1.55-1.79)	1.25(1.12-1.39)
Age, 70-79 years	2.73(2.39-3.11)	1.59(1.47-1.71)	2.71(2.36-3.11)	2.71(2.36-3.11)	2.24(2.07-2.43)	2.11(1.95-2.29)	2.04(1.9-2.19)	1.45(1.39-1.52)	2.62(2.46-2.79)	1.84(1.67-2.02)
Age, ≥80 year	5.42(4.77-6.17)	2.94(2.73-3.16)	5.52(4.83-6.32)	5.52(4.83-6.32)	4.42(4.09-4.77)	4.05(3.75-4.37)	3.51(3.27-3.77)	2.36(2.25-2.47)	5.1(4.8-5.43)	3.32(3.03-3.63)
CCI	1.2(1.18-1.21)	1.19(1.18-1.19)	1.21(1.2-1.22)	1.21(1.2-1.22)	1.31(1.3-1.32)	1.25(1.25-1.26)	1.35(1.34-1.37)	1.28(1.28-1.29)	1.31(1.3-1.32)	1.17(1.16-1.18)
Depression	0.85(0.79-0.91)	0.77(0.75-0.8)	0.82(0.76-0.89)	0.82(0.76-0.89)	0.77(0.73-0.81)	0.77(0.75-0.8)	0.77(0.72-0.82)	0.97(0.93-1.01)	0.8(0.77-0.84)	0.75(0.71-0.79)

COPD: chronic obstructive pulmonary disease. Liver disease: moderate or severe. Cancer: any malignancy, including metastatic solid tumour.

CCI: Charlson Comorbidity Index. Excluding diabetes and the condition analysed. OR: Odds Ratio.CI: Confidence interval.
